# Effect of modified laparoscopic hysterectomy on pelvic floor function: A retrospective observational study

**DOI:** 10.1097/MD.0000000000014616

**Published:** 2019-02-22

**Authors:** Chung-Yuan Lee, Chih-Jen Tseng, Chia-Hao Chang, Meng-Chih Lee, Yu-Che Ou, Shun-Fa Yang

**Affiliations:** a Institute of Medicine, Chung Shan Medical University, Taichung; b Department of Obstetrics and Gynecology, Chia-Yi Chang Gung Memorial Hospital; c Department of Nursing, Chang Gung University of Science and Technology, Chia-Yi Campus, Chia-Yi; d Department of Obstetrics and Gynecology, Chung Shang Medical University Hospital, Taichung; e College of Nursing & the Chronic Diseases and Health Promotion Research Center, Chang Gung University of Science and Technology, Chia-Yi Campus, Chia-Yi; f Department of Medical Research, Chung Shan Medical University Hospital, Taichung, Taiwan, R.O.C.

**Keywords:** laparoscopic colposuspension, laparoscopy-assisted vaginal hysterectomy, vaginal length

## Abstract

Hysterectomy is a potential risk factor for subsequent surgery for pelvic organ prolapse, especially when the prolapse exists before hysterectomy. Women without prolapse before hysterectomy may also experience prolapse after hysterectomy. This study aimed to describe a surgical modification of laparoscopic colposuspension with round ligaments after hysterectomy in women without preexisting genital prolapse and to evaluate the initial surgical results in these patients.

We reviewed data of 54 patients who underwent laparoscopic hysterectomy with colposuspension with unilateral or bilateral round ligaments after hysterectomy at Chia-Yi Chang Gung Memorial Hospital from July 2012 to March 2015. Vaginal length was measured before and after colposuspension after complete hysterectomy. Preoperative characteristics of the patients, perioperative quality, postoperative outcomes, and vaginal length differences were analyzed.

Vaginal length increased by a mean of 2.59 cm after colposuspension. The mean extra-operative time needed for laparoscopic colposuspension was about 10 minutes. No severe complications were reported in our patients, and we did not find any cystocele after completing vaginal cuff suspension to the round ligament.

The vaginal apex level was maintained in our modified laparoscopic hysterectomy. Therefore, laparoscopic colposuspension with round ligaments is a promising option as a routine, first-line standard procedure in younger women without genital prolapse to maintain an acceptable vaginal length after laparoscopic hysterectomy.

## 1 Introduction

Hysterectomy is a typical gynecological procedure that is performed for various benign diseases using abdominal (56%), vaginal (19%), laparoscopic (20%), and robotic (5%) approaches.^[1]^ In the US, the annual rate of hysterectomy peaked in 2002 at over 680,000 procedures performed; thereafter, this rate began decreasing, and in 2010, 430,000 inpatient hysterectomies were reported.^[2]^ The most common diagnostic indications for hysterectomy are as follows: uterine leiomyoma, pelvic organ prolapse, pelvic pain or infection (e.g., endometriosis and pelvic inflammatory diseases), abnormal uterine bleeding, and malignant and premalignant diseases.

The first laparoscopic hysterectomy was performed in 1989.^[3]^ Developments in gynecologic surgery have led to more minimally invasive options for hysterectomy. Less invasive procedures are typically preferable to more invasive procedures, where possible.^[4]^ The consensus for hysterectomy in benign disease suggests using minimally invasive techniques when appropriate and possible.^[5,6]^

Hysterectomy is known as a potential risk factor of subsequent surgery for pelvic organ prolapse, especially when the prolapse exists before hysterectomy, regardless of the surgical procedure performed. A large case-control study of 160,000 women who underwent hysterectomy showed that the risk of prolapse after hysterectomy was significant; however, this study did not illustrate whether prolapse was an indication for hysterectomy.^[7]^ Women without prolapse before hysterectomy may exhibit prolapse after hysterectomy, and the factors surrounding this progression are unclear. In 1 prospective study of 376 women, no association was found between previous hysterectomy for non-prolapse indications and surgery for pelvic organ prolapse or urinary incontinence.^[8]^ The possible mechanisms of post-hysterectomy prolapse include surgical damage in the connective tissue or injury to the innervation and vascularization of the pelvic floor muscles intraoperatively.

Therefore, preventing pelvic prolapse after hysterectomies performed for indications other than prolapse is important. The objectives of this study were to describe a surgical modification of laparoscopic colposuspension with round ligaments after hysterectomy and to evaluate the initial surgical results in women without preexisting genital prolapse.

## 2 Methods

A total of 54 patients underwent laparoscopic hysterectomy with colposuspension with unilateral or bilateral round ligaments after hysterectomy at Chia-Yi Chang Gung Memorial Hospital from July 2012 to March 2015. Inclusion criteria were as follows: benign disease, such as uterine myoma, adenomyosis, endometrial atypical complex hyperplasia, and cervical severe dysplasia, all of which are indications for laparoscopic hysterectomy. Exclusion criteria were as follows: diagnosed uterine and cervical malignancy, contraindications for treatment with laparoscopic surgery such as severe obesity, possibility of severe intra-abdominal adhesion, old age, malignancy risk, or a short residual round ligament unsuitable for colposuspension. Chang Gung Medical Foundation Institutional Review Board approved this study (approval no. 106.09.23), and informed consent was not required from the participants due to the retrospective nature of the research.

### 2.1 Surgical technique

The laparoscopic equipment used (Karl Storz SE&Co. KG, Tuttlingen, Germany) included a 19’ LCD surgical monitor, a Tricam camera control unit, a Tricam CCD camera head, a Xenon 300 light source, a thermoflator, and a 0-degree rigid scope.

Patients were placed in the dorsal lithotomy position while they were under general anesthesia. First, hysterectomy was performed using a 3-port laparoscopy-assisted vaginal hysterectomy (LAVH) or single-port laparoscopy-assisted vaginal hysterectomy (SILS-port LAVH) technique. For 3-port LAVH, we placed a 12-mm trocar (Covidien LLC, CT) in the umbilicus for the scope and 5-mm trocars (Lagis trocar, Taichung Taiwan) lateral to the rectus abdominis muscles, 2 cm above and 2 cm medial to the anterior superior iliac spine. For SILS-port LAVH, we placed a SILS port (Covidien LLC, CT) in the umbilicus, as previously described.^[9]^ The routine laparoscopic hysterectomy was considered complete after vaginal cuff closure, after which bleeding was checked and surgery completed. Before colposuspension was performed, we tested the tension by approximation of the vaginal cuff and round ligament. If the residual round ligament was short and the tension was such that there was a possibility or risk of tearing the tissue after colposuspension, we did not proceed with the procedure. Otherwise, colposuspension was then initiated.

**Figure 1:**
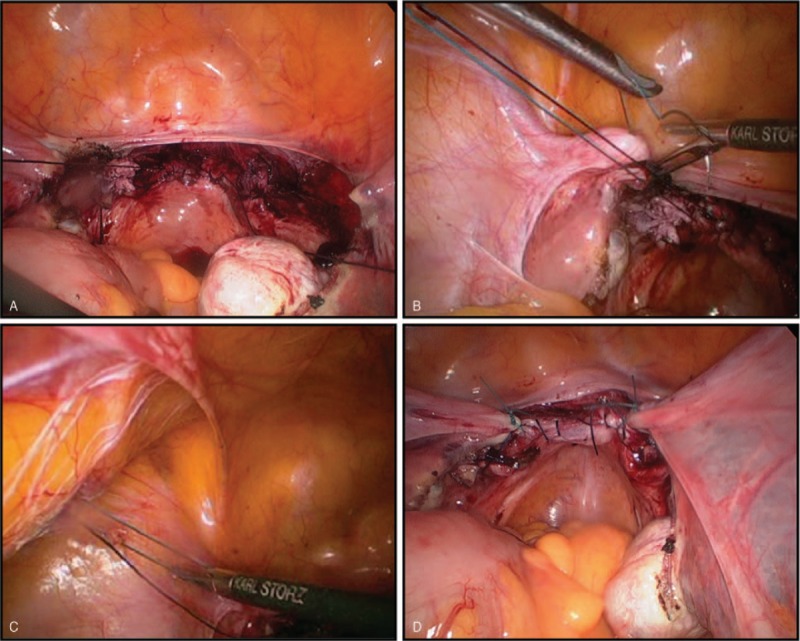
Laparoscopic bilateral vaginal cuff suspension with round ligament. (A) Two 1-0 Vicryl sutures are advanced into the pelvic cavity at the end of each side for traction after vaginal cuff closure. (B) Laparoscopic suturing is performed from the left vaginal stump to the left round ligament. (C) The knot is inserted into the pelvic cavity and advanced with assistance by the laparoscopic instrument. (D) Laparoscopic ligation of the round ligaments with a vaginal cuff on each side is completed.

The first step of bilateral vaginal cuff suspension was to advance two 1–0 Vicryl (VICRYL^TM^, Ethicon, Inc., Somerville, NJ) sutures into the pelvic cavity at the end of each side for traction after vaginal cuff closure (Fig. 1).

The 1–0-Vicryl vaginal cuff suture line on the left side was extracted from the pelvic cavity through a 5-mm trocar (the same procedure was performed on the right side). Traction forces on the left 1–0-Vicryl suture on the vaginal stump were used to estimate the required tension, and the suture point of left round ligament was approached. The 1–0 Vicryl, non-resolvable 1–0 Prolene (PROLENE^TM^, Ethicon, LLC., San Lorenzo, PR), or 2–0 Ethibond (ETHIBOND^TM^, Ethicon, Inc., Somerville, NJ) suture line was inserted into the abdomen through the trocar side and the other end of the suture remained outside of the abdomen.

The next step involved laparoscopically suturing from the left vaginal stump to the left round ligament (Fig. 1). An extracorporeal simple knot was created, and the knot was inserted into the pelvic cavity and the knot advanced with assistance by the laparoscopic instrument (Fig. 1). Then, the round ligament was cautiously approximated to the vaginal vault. After completing the first knot of colposuspension, 6 or more extracorporeal security knots were made to prevent loosening of the knot. The same was accomplished on the right side.

Finally, laparoscopic ligation of the round ligaments with a vaginal cuff on each side was completed (Fig. 1).

### 2.2 Vaginal length measurement pre- and post-laparoscopic colposuspension

We measured vaginal length from the vaginal apex to the vaginal orifice with the Levonorgestrel-Releasing Intrauterine System (LNG-IUS, Mirena, BAYER OY, Turku, Finland) outer sheath before and after colposuspension. All measurements were agreed to by participants in this study. Data were recorded by the operator, operation assistant, and scrub nurse.

### 2.3 Statistical analysis

All categorical and continuous variables were presented as percentages, and mean ± SD, and median (range), as appropriate. The paired *t* test was performed to assess the change in vaginal length. All *P*-values were obtained using 2-tailed tests. A *P* value less than .05 was considered statistically significant. SAS version 9.4 (SAS Institute Inc., Cary, NC) was used for statistical analysis.

## 3 Results

The preoperative characteristics of patient including the type of LAVH, whether the colposuspension involved unilateral or bilateral round ligaments and the indication of the surgery are shown in Table 1. All patients underwent laparoscopic hysterectomy by traditional 3-port or single-port laparoscopy and laparoscopic colposuspension with unilateral or bilateral round ligament. Table 2 displays the surgical characteristics and postoperative outcomes. Twelve patients had blood transfusion before operation due to anemia. One patient had an operative blood loss of 1100 mL due to a huge uterine myoma, but she was discharged at 3 days postoperatively.

**Table 1:**
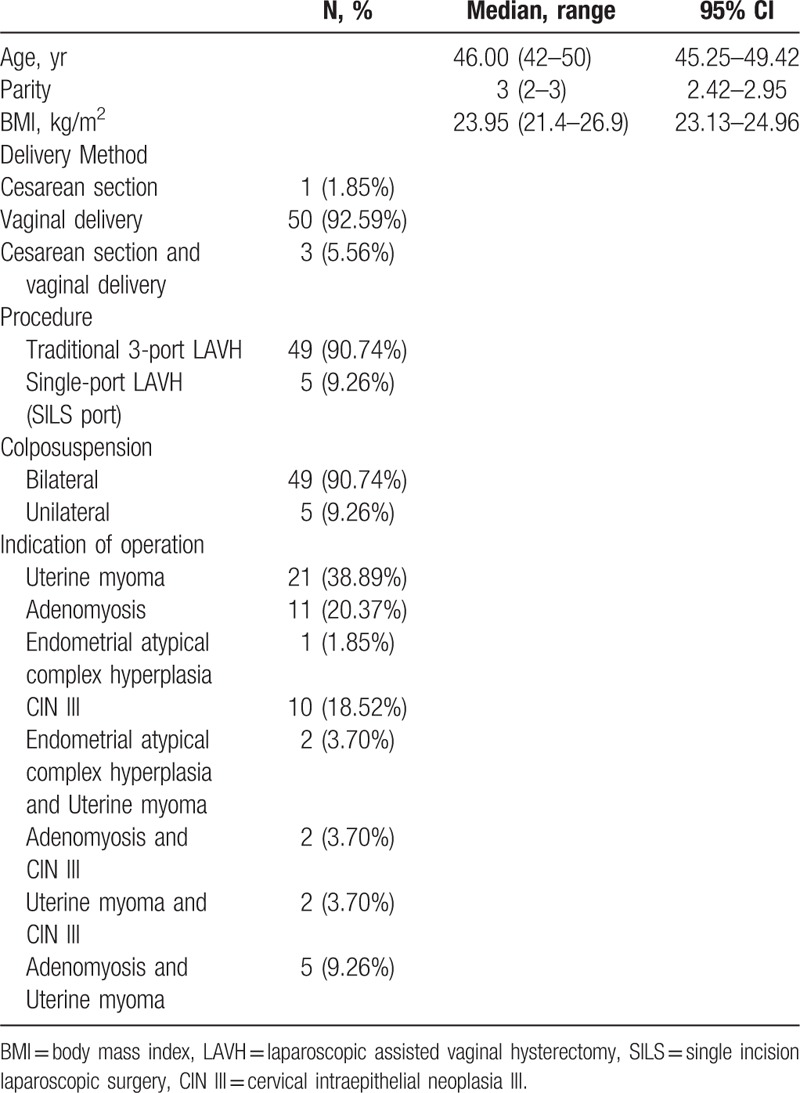
Basic information.

**Table 2:**
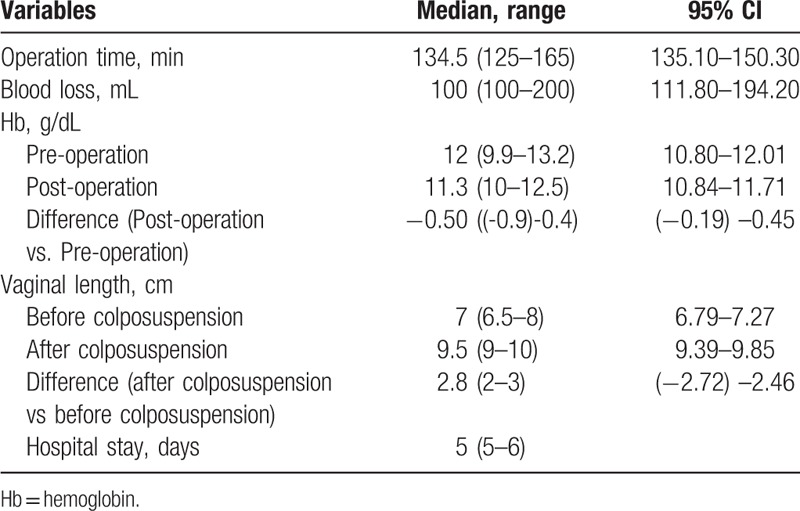
Surgery characteristics and postoperative outcomes.

**Figure 2:**
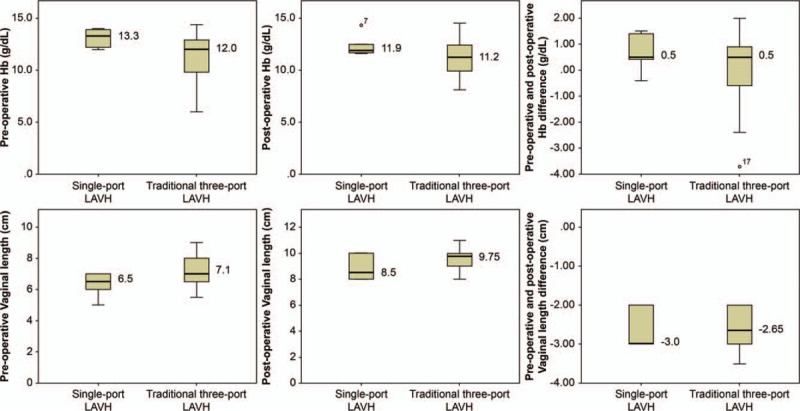
The median (range) of pre-operative Hb, post-operative Hb, post-operative vs. pre-operative Hb difference, vaginal length before colposuspension, vaginal length after colposuspension, and vaginal length difference before and after colposuspension via single-port LAVH and traditional 3-port LAVH. Hb =  hemoglobin,LAVH = laparoscopy-assisted vaginal hysterectomy.

After colposuspension, vaginal length increased significantly with a mean of 2.59 ± 0.47 cm (t = 40.27, *P* <.001; before, 7.03 ± 0.88 cm; after, 9.62 ± 0.83 cm). Figure 2 shows the median (range) of pre-operation Hb, post-operation Hb, post-operation vs. pre-operation Hb difference, vaginal length before colposuspension, vaginal length after colposuspension, and vaginal length difference before and after colposuspension via SILS-port LAVH and traditional 3-port LAVH. No complications were observed, including organ injury, hematoma, and postoperative infection. No cystocele was observed. We evaluated the vaginal length before and immediately after colposuspension.

## 4 Discussion

The benefits of laparoscopy are well known and include minimal invasiveness, quicker recovery, shorter hospitalization, and lower likelihood of adhesion. It can provide better avascular surgical field and it significantly reduces the likelihood of intraoperative and postoperative complications, such as massive bleeding, and nerve, ureteral, bowel, and bladder injuries, along with reducing blood loss.^[10,11]^ The objective of this study was to evaluate differences in vaginal length after colposuspension with unilateral or bilateral round ligaments after traditional 3-port or single-port laparoscopic hysterectomy in patients with indications for laparoscopic hysterectomy. The results indicate that laparoscopic unilateral or bilateral vaginal cuff fixed with round ligaments after hysterectomy is an easy procedure resulting in effective maintenance of vaginal length to prevent vaginal vault prolapse.

Women undergo hysterectomy to resolve many symptoms, such as menorrhagia, dysmenorrhea, and fibroid compression; however, vaginal vault or genital prolapse can occur after hysterectomy due to reduction or loss of natural support from the pelvic organs. If genital prolapse is confirmed before surgery, there is a higher possibility of reoperation for recurrent pelvic prolapse.^[12]^ Additionally, there may be a high rate of surgical failure, repeated surgery for recurrent prolapse, and incontinence after previous pelvic floor surgery.^[13]^ Therefore, prophylactic vaginal apical fixation is important. Many established surgical procedures for vaginal vault fixation have been previously described. Regarding surgical pelvic structure correction, there is no single technique that offers a complete solution for all cases.^[14]^

A meta-analysis with a median follow-up of 25 months reported that transvaginal uterosacral ligament suspension resulted in good apical support in 98% of patients.^[15]^ However, these procedures have complication rates as high as 11%, including ureteric injury and kinking.^[16]^ To ensure posterior vaginal support, sacrospinous ligament suspension is a viable option, but it increases the risk of recurrent or anterior compartment prolapse in 25% to 30% of patients.^[17]^ Less invasive vaginal surgery for pelvic organ prolapse seems to be technically difficult and has a high risk of recurrence.^[18]^

Recently, laparoscopic equipment has become more technically advanced and many surgeons possess the necessary skills to perform laparoscopy. An increasing number of patients are choosing laparoscopic hysterectomy. Laparoscopic surgery can be performed after hysterectomy for vaginal cuff suspension to prevent vaginal vault prolapse, even in the absence of genital prolapse. Conventional laparoscopic colposuspension can be performed by suspension to the round ligaments, uterosacral ligaments, or sacral promontory.^[19]^ In women with existing genital prolapse, laparoscopic colposuspension with bilateral round-infundibulopelvic ligaments,^[12]^ uterosacral ligament,^[14]^ and sacral colpopexy^[20–22]^ is reported to have good outcomes and low recurrence rates. Recently, an artificial mesh has been used for pelvic organ prolapse support; however, this carries the risk of complications, such as infection and erosion into the bowel or bladder. The mesh erosion rate in genital prolapse surgery after hysterectomy is 2.3%.^[23]^ Laparoscopic mesh suspension for pelvic organ prolapse seems to have a greater learning curve for surgeons due to the complexity of the procedure and the necessary laparoscopic suturing skills required by the surgeon, and this increases operation time. Patients may also be hospitalized for longer and this reduces the cost-effectiveness of the procedure.

Abdominal retropubic suspension can correct the anatomical anomaly by pulling the vault to the Cooper's ligament level to maintain vaginal length and reducing the likelihood of damage to the rectum and sciatic nerve.^[24–26]^ Kim et al^[12]^ introduced a post-hysterectomy method for high-grade uterovaginal prolapse through laparoscopic colposuspension with bilateral round-infundibulopelvic ligaments for women with existing genital prolapse. Yoon et al^[27]^ also performed chart reviews of 43 patients with uterovaginal prolapse who underwent laparoscopic colposuspension to the Cooper's ligament after hysterectomy. Regarding the maintenance of the vaginal length, we performed laparoscopic colposuspension to the round ligament in younger women without genital prolapse after hysterectomy and evaluated its effectiveness.

We aimed to design an easy, quick, and simple procedure without intracorporeal suturing to achieve fixation of the vaginal vault to the round ligament. The procedure showed reasonable operation time, blood loss, differences between preoperative and postoperative Hb, and postoperative duration of hospitalization. Additionally, we believe that our surgical approach has a smaller learning curve. The mean additional operative time spent in laparoscopic colposuspension in the present study was about 10 minutes. There were no severe complications, including postoperative internal bleeding, infection, and nerve, ureteral, bladder and bowel injuries, observed in our patients. The surgical approach also showed efficacy in maintaining the vaginal apex. We found no cystocele after completing vaginal cuff suspension to the round ligament. Our method increased vaginal length by a mean of 2.59 ± 0.47 cm after colposuspension.

Despite the advantages observed in our study, there were also several limitations. First, the number of cases was limited. Further studies with larger samples are needed to confirm our results. While this study evaluated the efficacy of vaginal length after colposuspension, this was in the immediate postoperative period, and therefore further studies are required to assess the efficacy of vaginal cuff suspension over the long term. In the future, we would like to follow these cases to ensure vaginal length maintenance and pelvic support. In addition, a further study based on the distribution of questionnaires for the assessment of longer-term postoperative satisfaction, sexual life satisfaction, and urinary symptoms, including incontinence and frequency, is warranted.

Among the types of laparoscopic colposuspension in women with existing pelvic organ prolapse, a laparoscopic vaginal cuff fixed with a round ligament may be less effective with a poorer success rate.^[19,28,29]^ However, this modified surgical procedure is easy, has a minimal learning curve, and results in immediate cystocele correction and minimal complications compared to more complicated laparoscopic colposuspensions for maintaining vaginal length after hysterectomy for women who are younger without prolapse. Laparoscopic colposuspension with round ligament may not be suitable for women with existing genital prolapse to correct pelvic organ prolapse after laparoscopic hysterectomy, but it shows potential as a routine, first-line, and standard procedure in younger women without genital prolapse to maintain acceptable vaginal length after laparoscopic hysterectomy.

## Acknowledgments

The authors are grateful to Dr Yi-Lun Chen for helpful discussions and table creation. We are also grateful to the Chang Gung Medical Foundation Institutional Review Board who approved the study.

## Author contributions

**Conceptualization:** Chung-Yuan Lee, Chih-Jen Tseng, Shun-Fa Yang.

**Data curation:** Chung-Yuan Lee.

**Formal analysis:** Chung-Yuan Lee, Chia-Hao Chang.

**Funding acquisition:** Shun-Fa Yang.

**Investigation:** Chung-Yuan Lee, Chih-Jen Tseng, Chia-Hao Chang, Meng-Chih Lee, Yu-Che Ou, Shun-Fa Yang.

**Methodology:** Chung-Yuan Lee, Chih-Jen Tseng.

**Project administration:** Chung-Yuan Lee.

**Resources:** Chung-Yuan Lee, Yu-Che Ou, Shun-Fa Yang.

**Software:** Chia-Hao Chang.

**Supervision:** Meng-Chih Lee, Yu-Che Ou, Shun-Fa Yang.

**Validation:** Chung-Yuan Lee, Chih-Jen Tseng, Chia-Hao Chang, Meng-Chih Lee, Yu-Che Ou, Shun-Fa Yang.

**Visualization:** Chung-Yuan Lee.

**Writing – original draft:** Chung-Yuan Lee.

**Writing – review & editing:** Chung-Yuan Lee, Chih-Jen Tseng, Meng-Chih Lee, Shun-Fa Yang.
